# Non traumatic cataract dislocation: when to seek an etiology?

**DOI:** 10.11604/pamj.2014.19.73.5016

**Published:** 2014-09-24

**Authors:** Othman Charhi, Rajae Daoudi

**Affiliations:** 1 Mohammed V University Souissi, Faculty of Medicine, Department “A” of Ophthalmology, Rabat, Morocco

**Keywords:** Cataract dislocation, etiology, Marfan syndrome

## Image in medicine

A 27 years old woman without medical or surgical records, consults for a bilateral low vision since her childhood, the exam found a visual acuity of 3/10 on the right and left eye. The slit lamp exam found a bilateral cataract. After a dilatation with tropicamid; we discovered a superior-temporal lens dislocation in both eyes. The three major etiologies of non traumatic lens dislocation with or without a cataract are: Marfan syndrome, Weill Marchesani syndrome and Homocystinury. The Marfan syndrome is the most frequent and represents 40% of all congenital lens dislocation. It's a general disease that affects the metabolism of the collagen of all the body that is weak. This can manifest with lens dislocation in young patient generally in superior and temporal side due of the weakness of the zonula that brokes. It can be accompanied with physical abnormalities like tallness; hail members and ligamentar over laxity. Cardiovascular associated abnormalities like aortal aneurysm represents the major danger of this disease because it can evolve to an aortic dissection of and lead to sudden death. In our case, the length of our patient was 172 cm. The cardiovascular exam didn't show any abnormalities. The patient went under surgery and benefit from a phacophagia of both cataracts.

**Figure 1:**
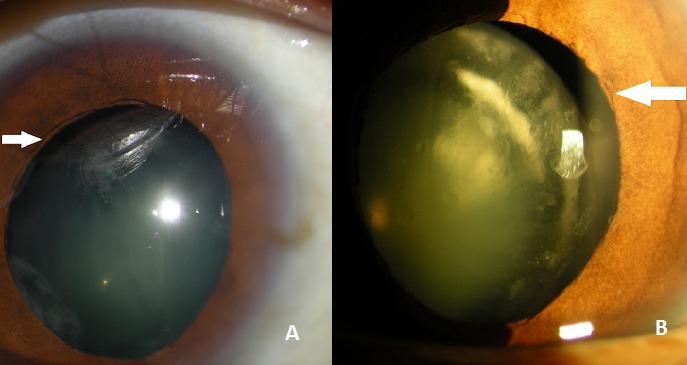
A) right eye showing the cataract dislocation and the zonular rupture (white arrow); B) left eye showing the cataract dislocation in the superior temporal side with zonular rupture (white arrow)

